# Optimizing spatial accessibility and equity of hierarchical older adult care facilities using a multi-modal two-step floating catchment area method: a case study of Lin'an District, Hangzhou

**DOI:** 10.3389/fpubh.2025.1559463

**Published:** 2025-04-23

**Authors:** Mengxia Gu, Shangbo Li, Guoquan Zheng

**Affiliations:** ^1^College of Landscape Architecture, Zhejiang A&F University, Hangzhou, China; ^2^School of Civil Engineering and Architecture, Zhejiang University of Science and Technology, Hangzhou, China

**Keywords:** older adult service facilities, two-step mobility search method, spatial accessibility, multiple trips, multiple tiers

## Abstract

The global aging trend is becoming increasingly pronounced, and the accessibility and equity of older adult care facilities directly influence the health and quality of life of the older adult population, thus representing a critical issue in public health research and policy-making. Using Lin'an District, Hangzhou as an illustrative case, this research examines urban-rural integrated areas specifically, addressing the persistent challenge of supply-demand mismatches in older adult care facility allocation and seeking to optimize their spatial configuration. A comprehensive analytical framework based on the multi-modal two-step floating catchment area (2SFCA) method was established, integrating the Gini coefficient, Lorenz curve, and local spatial autocorrelation analysis to systematically evaluate the spatial accessibility and equity of older adult care facilities. The results demonstrate significant spatial heterogeneity in facility accessibility, revealing a clear distribution pattern characterized by higher accessibility in the eastern urban core and markedly lower accessibility in western rural regions, thereby highlighting notable supply-demand imbalances between urban and rural contexts. Furthermore, the application of local spatial autocorrelation effectively identified key regions characterized by pronounced inequities, notably rural areas in the west suffering severe resource deficiencies and transitional urban-rural zones where supply-demand conflicts prominently occur. The study further investigates critical factors underlying accessibility and equity disparities, including differences in transportation infrastructure, uneven older adult population distributions, and hierarchical classifications of service facilities. Ultimately, the findings provide valuable empirical insights and policy recommendations applicable to urban-rural integration contexts globally, contributing meaningfully to the advancement of age-friendly societies.

## 1 Introduction

Global life expectancy continues to increase, and rapid urbanization is exacerbating the issue of population aging globally (1). Within this context, frameworks such as “aging in place” and “livable communities” have become prominent lenses for examining spatial equity and eldercare provision. Numerous countries have adopted age-friendly strategies in response: for instance, Japan's super-aged society emphasizes compact urban form to enhance walkability (2), while the European Union focuses on improving rural accessibility through multi-modal transportation systems (3). In China, population aging is closely linked to the rapid integration of urban and rural areas. Given that China's natural population growth rate turned negative in 2022 and individuals aged 60 or above reached one-fifth of the total population, the phenomenon of “longevity without health” has intensified the urgent demand for diverse older adult care services. Although the Chinese government has implemented policies to expand and standardize senior care facilities, significant discrepancies between supply and demand persist. Consequently, optimizing these services to meet heterogeneous needs has become a pressing challenge.

Accessibility has emerged as a fundamental metric for evaluating the spatial rationality and service efficiency of public facilities, aligning with the “livable communities” approach that emphasizes equitable access (4). Scholars both domestically and internationally have extensively investigated the accessibility of public service facilities, including medical institutions (5), educational institutions (6), parks (7), and green spaces, with increasing attention being paid to older adult service facilities. Initial studies primarily emphasized facility quantity, scale, service levels, and the number of beds (8–10). As research has progressed, accessibility has emerged as a crucial perspective for evaluating the equity of senior care facility layouts. For example, Li et al. (11) examined Chengdu as a case study to analyze spatial planning related to senior care institutions in megacities. However, existing studies on transportation modes predominantly focus on railroad networks at the macro level and modes such as walking, bus transit, and driving within cities. Yet, these studies are often limited to single travel modes or broad regional units, making it difficult to comprehensively capture the nuanced impact of diverse travel modes on older adult accessibility or reveal spatial disparities at the community or village level (12–15). Commonly employed methods for accessibility measurement include proximity analysis (16), potential models (11), buffer zone analysis (17), and the two-step floating catchment area (2SFCA) method (18). Among these, potential models and the 2SFCA method are widely utilized due to their ability to incorporate interactions between spatial distribution and supply-demand points. Compared to potential models, the 2SFCA method can visually depict supply-demand ratios, thereby facilitating optimized resource allocation. For instance, Chen et al. (19) applied the 2SFCA method to study park and green space accessibility in Hefei City, providing guidance for optimizing urban green space and rail transportation layouts. Despite its strengths, the traditional 2SFCA method has limited capacity to incorporate diverse transportation modes and travel times, constraining its accuracy. This study addresses this limitation by enhancing the 2SFCA method through incorporation of a Gaussian decay function and integration of multiple travel modes, including walking, public transportation, and driving, utilizing real-time traffic data obtained from network maps. These methodological enhancements significantly improve computational accuracy and broaden applicability, making them particularly suited for assessing older adult service facility accessibility in urban-rural combined areas. Thus, this approach offers novel insights into resource allocation disparities between urban and rural settings, as well as their underlying causes.

Equity and accessibility are intrinsically interconnected, jointly reflecting the rationality and balance of public service facilities in terms of spatial configuration and service provision (11, 20). Evaluations of equity in older adult service facilities mainly examine the ease with which older adult populations can access resources within reasonable distances, thus serving as a vital criterion for evaluating facility allocation quality. Existing equity analyses predominantly focus on the spatial equilibrium of facilities, often employing methods such as the Gini coefficient and Lorenz curve to effectively illustrate resource concentration and supply-demand matching (21). For example, Cheng et al. (22) assessed the fairness of the spatial distribution of senior care facilities using the Lorenz curve across different regions. Additionally, some scholars (7, 23) have applied kernel density analysis, spatial autocorrelation models, and logistic regression models to provide a refined understanding of equity and spatial distribution. However, these studies often limit their scope to urban or township scales, failing to capture equity characteristics at the finer community level (11, 24). Two major deficiencies currently exist in equity research methodologies and scope concerning older adult service facilities: firstly, comprehensive analyses integrating equity and accessibility remain scarce, with most studies addressing these aspects separately and lacking systematic analysis of their interactions. Secondly, existing research on spatial equity inadequately considers the complexity of transportation networks and the diverse needs for older adult services in urban-rural integration areas, especially in combined urban-rural contexts. Equity research methodologies are predominantly designed for urban environments, thus inadequately addressing the complex spatial patterns and resource distribution characteristics of urban-rural areas. This study aims to comprehensively examine accessibility and equity of older adult service facilities at the community scale, building upon accessibility research by integrating the Lorenz curve, Gini coefficient, and spatial autocorrelation methods to overcome existing research limitations. By elucidating the causes underlying resource allocation disparities and supply-demand imbalances in urban-rural integration contexts, this study provides robust scientific support for optimizing resource allocation.

Hangzhou was among China's first cities to enter an aging society, and Lin'an District exhibits the highest degree of aging among its municipal districts, characterized by a population structure intertwined with urban and rural characteristics. The socio-economic context shaped by rapid urbanization and ongoing rural integration contributes significantly to the varied older adult service demands. In recent years, Lin'an District has accumulated substantial practical experience in older adult facility construction and policy-driven optimization of urban and rural resource allocations. The district has established a comprehensive older adult service system and introduced the innovative “1+N” older adult service model. The district's unique urban-rural socio-economic characteristics shape diverse older adult care demands, providing valuable insights and practical lessons for policy-level optimization.

**Figure 1:**
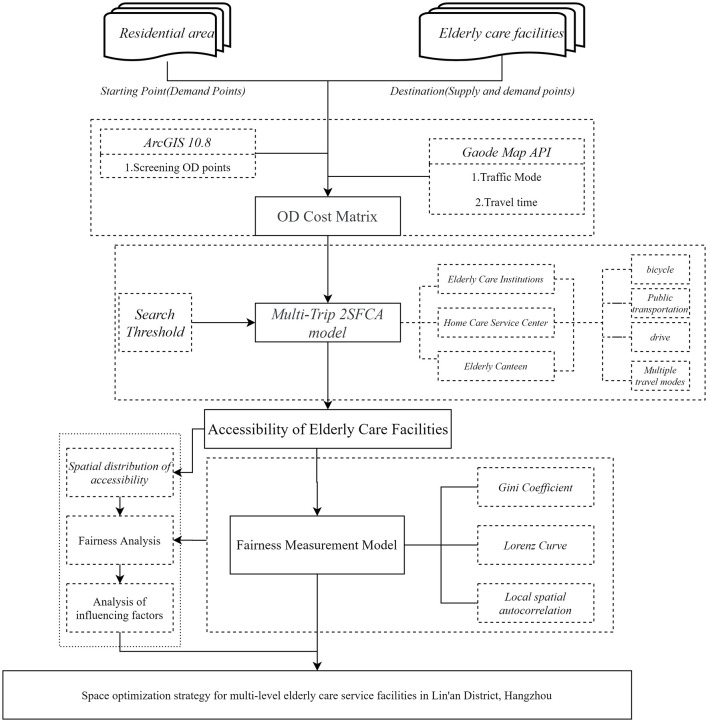
Technology roadmap.

The current study employs a methodological framework integrating Python-based web scraping to obtain and clean POI and AOI data, constructing origin-destination tables, and retrieving real-time traffic data through an open map API. Multiple travel modes—walking, public transportation, and driving—are incorporated into an enhanced “two-step mobility search method” utilizing a Gaussian decay function. Equipped with improved accuracy, this approach highlights community-level imbalances and disparities between supply and demand. The research offers evidence-based insights and practical policy recommendations for developing age-friendly urban and rural environments. The findings possess broader implications for rapidly urbanizing regions facing similar aging challenges, emphasizing the importance of integrating accessibility and equity considerations into eldercare planning (Figure 1).

## 2 Materials and methods

### 2.1 Study area

**Figure 2:**
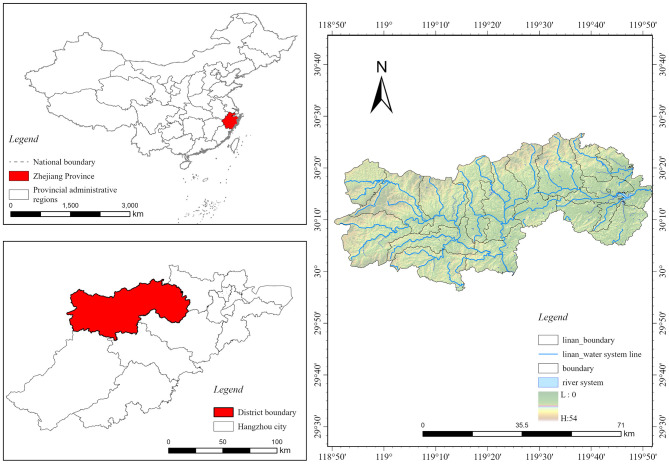
Lin'an District location map.

**Figure 3:**
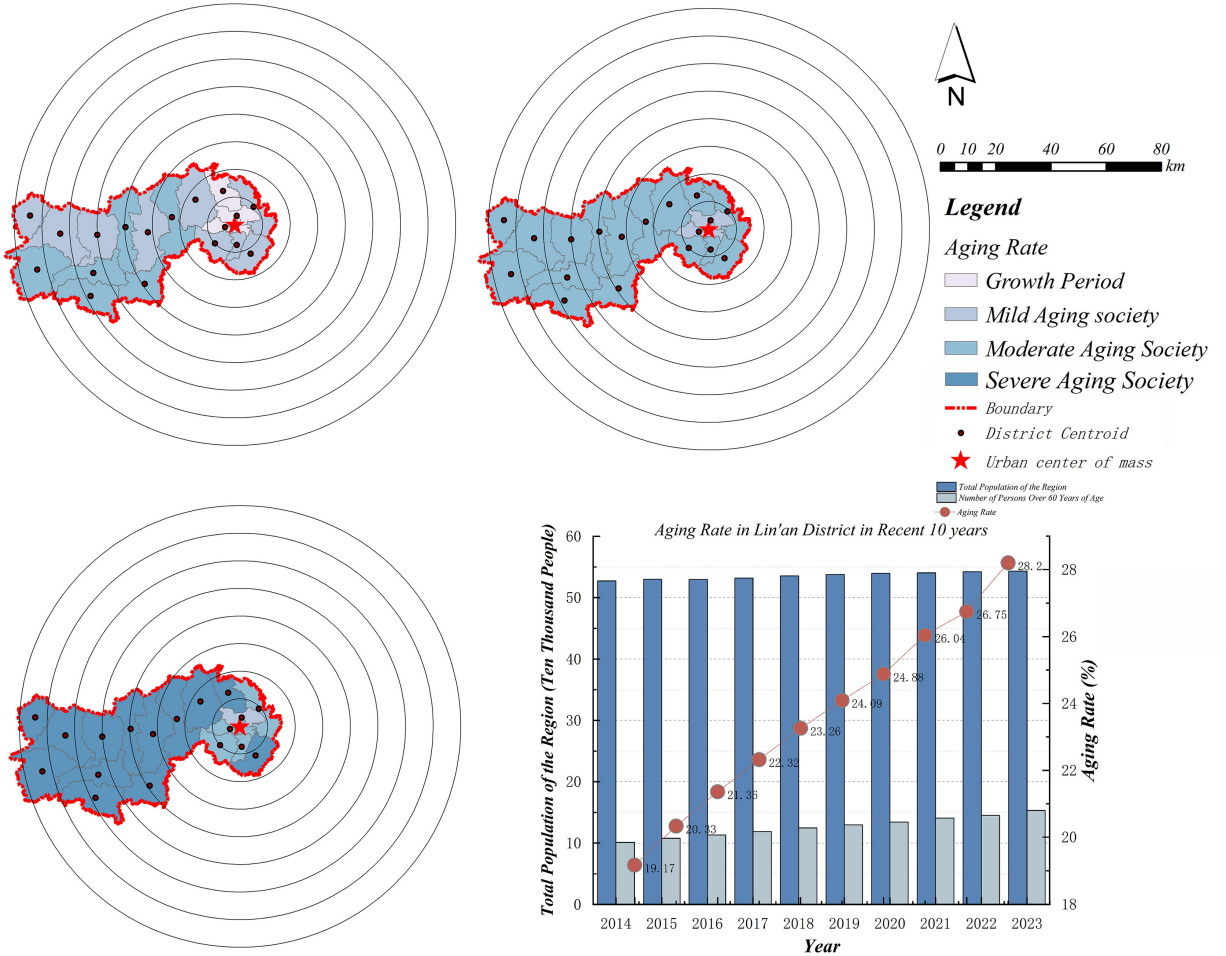
Spatial and temporal changes in aging rates.

Lin'an District is located in the western part of Hangzhou City, Zhejiang Province (Figure 2), characterized by a topography that slopes from northwest to southeast, surrounded by mountainous terrain. It covers a total area of 3,126.8 square kilometers and administratively comprises five streets, including Jincheng, Jinbei, and Jinnan, and 13 townships, such as Banqiao Township and Gaohong Township. As of 2023, the district had an estimated resident population of 652,000, among whom 28.2% were aged 60 and above, making it the district with the highest aging level in Hangzhou. According to the United Nations' Classification Criteria on Aging, Lin'an District qualifies as a severely aging society. Additionally, over the past decade, the spatial distribution of aging within the district has exhibited a distinctive concentric pattern (Figure 3), with the aging rate progressively rising from the central urban area toward the periphery. Such spatial agglomeration of older adult populations significantly intensifies pressure on older adult care services.

### 2.2 Data processing

Older adult service facilities deliver crucial support, providing essential services including housing assistance, healthcare provision, and social engagement activities. These foundational services significantly contribute to older adult wellbeing. This research systematically categorizes older adult service facilities in Lin'an District by integrating demand-side characteristics, supply-side capacities, and underlying foundational data. Specifically, facilities offering core functions such as residential care, daily life assistance, cultural engagement, and healthcare are comprehensively evaluated through their spatial distributions, employing a classification method derived from the integration of point-of-interest (POI) and area-of-interest (AOI) data extracted from AMap via Python. The established classification explicitly reflects older adult population demands, combining spatial and demographic dimensions to facilitate a thorough assessment of accessibility.

Traditional methods of data collection typically depend on government-released statistical datasets, which, despite their reliability, often exhibit limited availability and infrequent updates. To address these limitations, this study employs Internet-based big-data techniques, significantly enhancing data timeliness and comprehensiveness (Table 1). Data were consistently collected during typical operational hours (8:00–18:00) to ensure representativeness. To further ensure data reliability, this study conducted a rigorous two-step verification process for real-time travel data obtained from the AMap API. First, API-derived travel time data were compared with results from on-site field surveys, demonstrating a high level of consistency. Second, API travel times were cross-referenced with publicly available transportation reports, further validating their accuracy and robustness.

**Table 1:** Specific details of data used in the research.

**Level**	**Data type**	**Data source**	**Data acquisition time**
***Demand-level***	*Street and village older adult population data*	*Sixth and seventh census data*	*2020.12*
		*Bureau registered population data*	*2024.7*
		*Statistical data of Lin 'an District*	*2023.8*
***Supply-level***	*Older adult care service facility data*	*Older adult care service facilities information*	*2024.8*
		*AMap POI data*	*2024.8*
		*AMap path planning data*	*2024.9*
***Base-level***	*Terrain data, road network data, area data, etc*.	*AMap AOI data crawling*	*2024.7*

**Figure 4:**
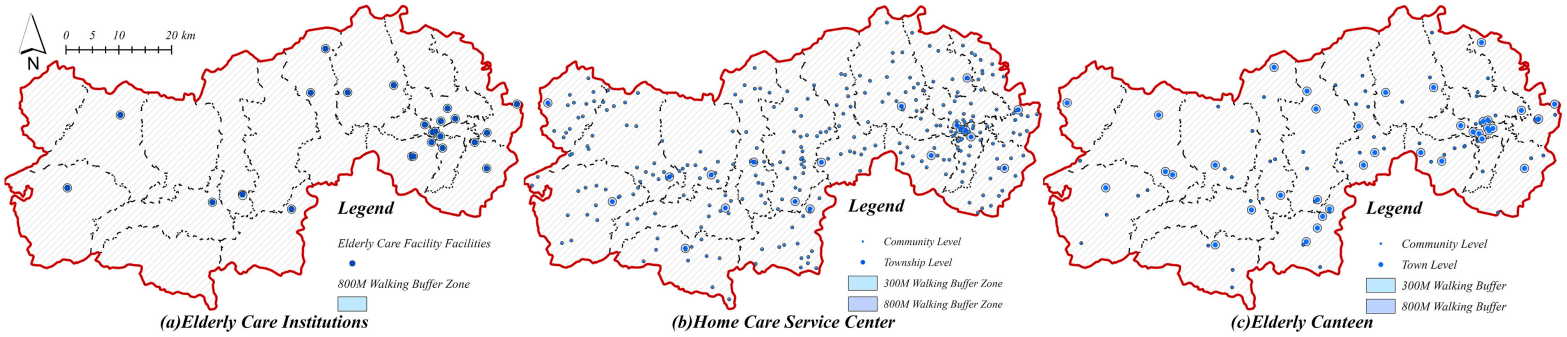
Map of the distribution and buffer zones of older adult service points. **(a)** Older adult care institutions. **(b)** Home care service center. **(c)** Older adult canteen.

Additionally, the research strictly adhered to the Provisions on Supporting Public Service Facilities for Territorial Spatial Planning (Figure 4), processing official community-level older adult population statistics into 600 m × 600 m spatial raster units to enhance analytical precision. Integrating these refined raster datasets into a comprehensive multi-modal 2SFCA accessibility model incorporating Gaussian distance decay, the analysis precisely aligns older adult care facility supplies with actual population demands. This methodological advancement provides a robust framework for assessing facility supply-demand balances in urban-rural integrated contexts, thereby delivering valuable empirical insights and substantially enhancing policy relevance.

### 2.3 Methods

This study employs multiple spatial statistical techniques to systematically evaluate the equity in the spatial allocation of older adult service facilities within Lin'an District. It begins by examining the spatiotemporal dynamics of the older adult population at the township level, emphasizing demographic shifts and their spatial distributions over time. The radius of service coverage is systematically determined by integrating older adult population density data into an enhanced Gaussian two-step floating catchment area (Ga2SFCA) model, allowing for a detailed analysis of accessibility that accounts for facility categories, transportation modes, and geographical contexts. Furthermore, the study rigorously quantifies allocation fairness through the application of the Gini coefficient and Lorenz curve, providing both objective measurement and intuitive visual representation of spatial equity patterns. Additionally, spatial autocorrelation analysis (Moran's I) is conducted to pinpoint areas exhibiting concentrated spatial inequities, effectively integrating accessibility and equity evaluations across multiple spatial dimensions, thereby enabling a comprehensive understanding of facility distribution fairness.

#### 2.3.1 Older adult service supply index

In order to systematically and quantitatively evaluate the service provision levels of older adult care facilities, this study employs Currie's (25, 26) evaluation methodology to establish an older adult service supply index, which is expressed through the following formula (Equations 1–3)


(1) $$\begin{array}{l} Sj=\overset{n}{\underset{i=1}{\sum }}\left(CI_{i}\times SL_{i}\right) \end{array}$$

(2) $$\begin{array}{l} CI_{i}=\frac{A_{Bi}}{A_{SD}} \end{array}$$

(3) $$\begin{array}{l} SL_{i}=\frac{M_{i}}{M_{0}} \end{array}$$

*I* represents a specific category of senior living service facility, *n* denotes the number of walking buffers within the community, and *S*_*j*_ denotes the community-level index of senior living service provision. To facilitate standardized quantitative comparison, we have normalized this index. *CI*_*i*_ represents the service coverage ratio of facility *i*, calculated as the proportion of the facility's walking buffer area within the community relative to the total area of the community. Based on the established travel threshold table (Table 2), various walking buffer levels are defined for different types of senior living service facilities. *SL*_*i*_ denotes the service level index of facility *i* defined as the ratio of the scale of a specific type of facility *M*_*i*_ to the total scale of facilities of the same type *M*_0_. In this context, *M*_*i*_ generally refers to the facility's floor area, the number of beds, and its official star rating (25, 26).

**Table 2:** Travel thresholds of older adult care service facilities.

	**Walking**		**Public transport**		**Driving**	
	***Community-level***	***Street-level***	***Community-level***	***Street-level***	***Community-level***	***Street-level***
*Older adult-care tnstitution*	*-*	*800 M*	*-*	*30 min*	*-*	*30 min*
		*15 min*				
*Home-care service-center*	*300 M*	*800 M*	*30 min*	*-*	*30 min*	*-*
	*5 min*	*15 min*				
*Older adult-canteen*	*300 M*	*800 M*	*30 min*	*-*	*30 min*	*-*
	*5 min*	*15 min*				

#### 2.3.2 Improved Gaussian two-step moving search method

The two-step search-movement method was originally proposed by Radke and Mu (27), aiming to evaluate spatial accessibility by separately analyzing the locations, scales, and demands of service supply and population demand points. It conducts two sequential searches within a predefined service radius to calculate accessibility based on cumulative supply-demand ratios. However, in practical settings, older adult individuals' demand for service facilities tends to diminish with increasing travel distance. To address this limitation, this study integrates the two-step search-movement method with a Gaussian attenuation function and incorporates three primary modes of older adult transportation—walking, public transit, and driving—thus establishing a multi-modal two-step search-movement method. This enhancement significantly improves the accuracy and precision of accessibility measurement (13, 28).

Initially, based on questionnaire surveys, field interviews, the official government rating system for various older adult care service facilities, relevant policy documents, and service delivery capacities, this study assigns specific weight coefficients to three categories of older adult service facilities. This weighting scheme enables systematic and comprehensive accessibility calculations, as well as clear visualization across different travel modes. Specifically, older adult care institutions were assigned a weight coefficient of 0.4, while home-based older adult care service centers and senior canteens were each assigned a weight of 0.3. Subsequently, accessibility for each facility type is calculated based on the travel mode proportions presented in Table 3, thereby obtaining the multi-modal accessibility metrics for each category of older adult service facility. Finally, these individual accessibility metrics are aggregated, resulting in the overall comprehensive multi-modal accessibility across Lin'an District. The specific computational formula is as follows (Equations 4–7):


(4) $$R_{j}=\frac{s_{j}}{\sum_{m\in M}\sum_{i\in \left\{t_{ij,m}\leq t_{0,m}\right\}}\left(P_{i,m}\times G_{t_{ij,m},t_{0,m}}\right)}$$

(5) $$G_{t_{ij,m},t_{0,m}}=\{\begin{array}{ll} \frac{e^{-1/2\times {\left(t_{ij,m}/t_{0,m}\right)}^{2}}-e^{-1/2}}{1-e^{-1/2}} & \text{ if }t_{ij,m}⩽t_{0,m}\\ 0 & \text{ if }t_{ij,m}>t_{0,m} \end{array}$$

(6) $$\begin{array}{l} A_{i,m}=\underset{j\in \left\{t_{ij,m}\leq t_{0,m}\right\}}{\sum }\frac{P_{i,m}\times R_{j}\times G_{t_{ij,m},t_{0,m}}}{\sum_{m\in \text{M}}P_{i,m}} \end{array}$$

(7) $$\begin{array}{l} A_{i}=\underset{m\in M}{\sum }A_{i,m} \end{array}$$

Where *R*_*j*_ is the ratio of demand and supply of older adult services at point *j*; *M* is three different modes of travel, *P*_*i,m*_ denotes the population size of older adult people traveling from point *i* to point *j* using travel mode *m*; *t*_*ij,m*_ denotes the travel time within the search radius; *t*_0,*m*_ is the travel threshold; *A*_*i,m*_ is the accessibility of a certain type of older adult service based on a certain travel mode *m*; *A*_*i*_ is the older adult service accessibility at *i*.

**Table 3:** The proportion and search radius of the older adult population in the three travel modes of older adult care service facilities.

**Mode of travel**	**Walking**	**Public transport**	**Driving**
*Proportion of the older adult population*	*63.20%*	*20.50%*	*16.30%*
*Search radius t_0_ (maximum value)*	*15 min*	*30 min*	*30 min*

#### 2.3.3 Traditional 2SFCA versus improved 2SFCA

The 2SFCA method, initially developed by Radke and Mu (27), is widely applied to evaluate the accessibility of public facilities. It does not, however, fully reflect the hierarchical nature of older adult care facilities, in which higher-tier institutions generally provide more comprehensive services and consequently attract residents from larger geographic areas. Additionally, the conventional 2SFCA method inadequately addresses distance-decay effects on older adult travel willingness, resulting in accessibility assessments that deviate from actual usage patterns. To overcome these limitations, the present study integrates multi-modal travel behaviors and a Gaussian distance-decay function into the 2SFCA method, comprehensively considering factors such as facility service capacity, hierarchical attractiveness, and older adult individuals' willingness to travel. This refined approach significantly enhances the accuracy and scientific rigor of accessibility evaluations for older adult care facilities.

**Figure 5:**
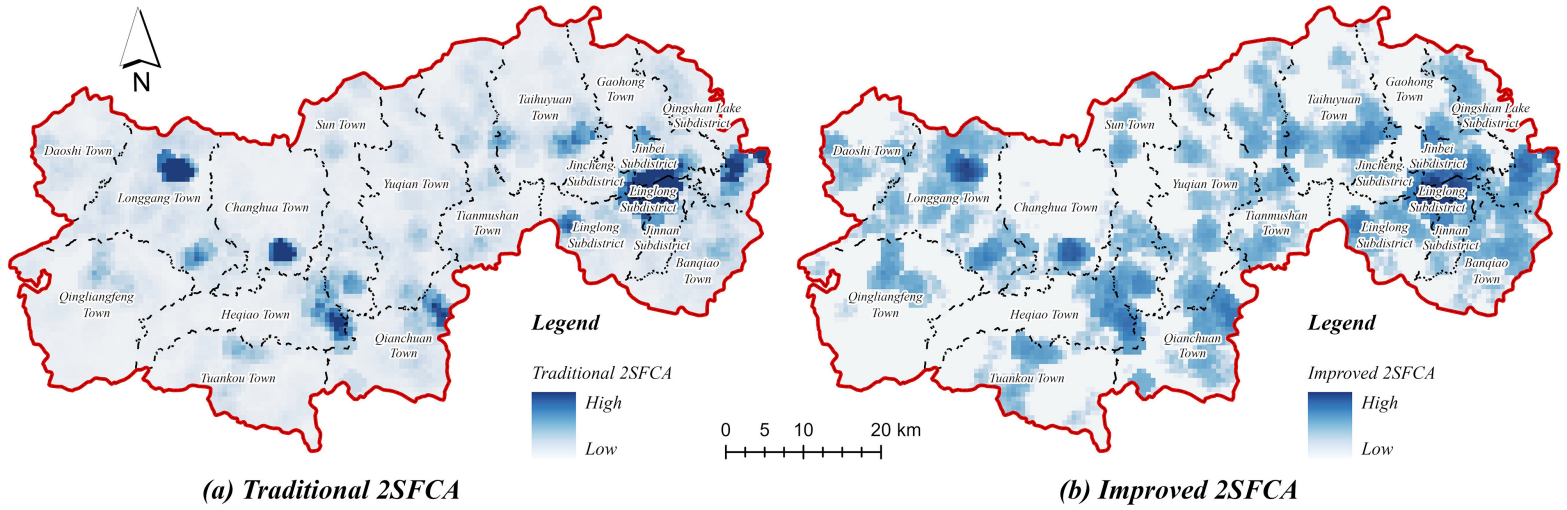
Evaluating walking accessibility to older adult care facilities in Lin'an district. **(a)** Traditional 2SFCA. **(b)** Improved 2SFCA.

Compared to the traditional 2SFCA method, the improved multi-modal 2SFCA method yields a more nuanced spatial distribution and more accurate accessibility estimates. As shown in Figures 5a, b the traditional method produces pronounced polarization in accessibility values, clearly delineating high- and low-accessibility zones. Conversely, the improved 2SFCA method demonstrates a more continuous and realistic transition within areas of high accessibility, facilitating finer intra-regional differentiation. Moreover, by incorporating facility hierarchy and travel willingness, the enhanced method accurately identifies areas such as community centers, which traditional methods misrepresent as low-accessibility zones, thus aligning more closely with actual spatial patterns. From a quantitative perspective, both methods exhibit significant gaps between maximum and minimum accessibility values (Table 4), highlighting prominent spatial disparities in older adult service resource allocation. However, by incorporating hierarchical attractiveness and distance decay effects, the improved method substantially narrows unrealistic extremes, offering a standardized range of accessibility values that better reflects real-world conditions. Consequently, the enhanced multi-modal 2SFCA method provides a more scientifically robust and practically meaningful assessment framework, effectively capturing intra-regional accessibility variations and the nuanced travel preferences of older adult populations.

**Table 4:** Comparisons of the spatial accessibility based on the two methods.

	***Minimum***	***Maximum***	***Average***	***Standard deviation***
Traditional 2SFCA	*0.0000*	*33.9019*	*0.4441*	*5.1399*
Improved 2SFCA	*0.0000*	*33.9209*	*0.4427*	*5.1385*

#### 2.3.4 Service capacity equity measurement model

The Gini coefficient quantifies equity as an integrated measure represented by a singular numeric value, effectively reflecting the overall distribution balance of older adult service resources (Table 5). Complementarily, the Lorenz curve visually depicts the equity of spatial distributions, clearly illustrating the proportional relationship between facility supply and older adult population demand. In this study, Gini coefficients are calculated for each type of older adult care facility as well as for the entire Lin'an District, and results are visualized through Lorenz curves, thereby enabling a comprehensive evaluation of supply-demand alignment and spatial equity. The calculation formula for the Gini coefficient is expressed as follows (Equations 8–9) (29):


(8) $$\begin{array}{l} G=1-\overset{n}{\underset{k=1}{\sum }}\left(P_{k}-P_{k-1}\right)\left(R_{k}+R_{k-1}\right) \end{array}$$

(9) $$\begin{array}{l} R_{k}=\frac{\sum_{i=1}^{k}A_{i}r_{i}}{\sum_{i=1}^{n}A_{i}r_{i}} \end{array}$$

Where *n* is the total number of grids and *k* is the number of settlements aligned. *P*_*k*_ is the demand capacity of the older adult population. *P*_0_ = 0. *P*_*n*_ = 1; *R*_*k*_ is to denote the ratio of accessibility to the cumulative older adult population from 1 to *k*. *R*_0_ = 0. *R*_*n*_ = 1; *r*_*i*_ is the population of settlement *i*.

**Table 5:** Gini index of resource allocation of older adult care service facilities.

**Gini index *(G)***	**Allocation of facilities for the aged**
***G** **≤0.2***	*Absolute fairness*
***0.2** **<** **G** **≤0.3***	*More fair*
***0.3** **<** **G** **≤0.4***	*Relatively reasonable*
***0.4** **<** **G** **≤0.6***	*The distribution is less reasonable*
***G** **>** **0.6***	*The distribution is grossly unreasonable*

## 3 Results

### 3.1 Spatial distribution of accessibility

#### 3.1.1 Accessibility of institutions for the older adult

**Figure 6:**
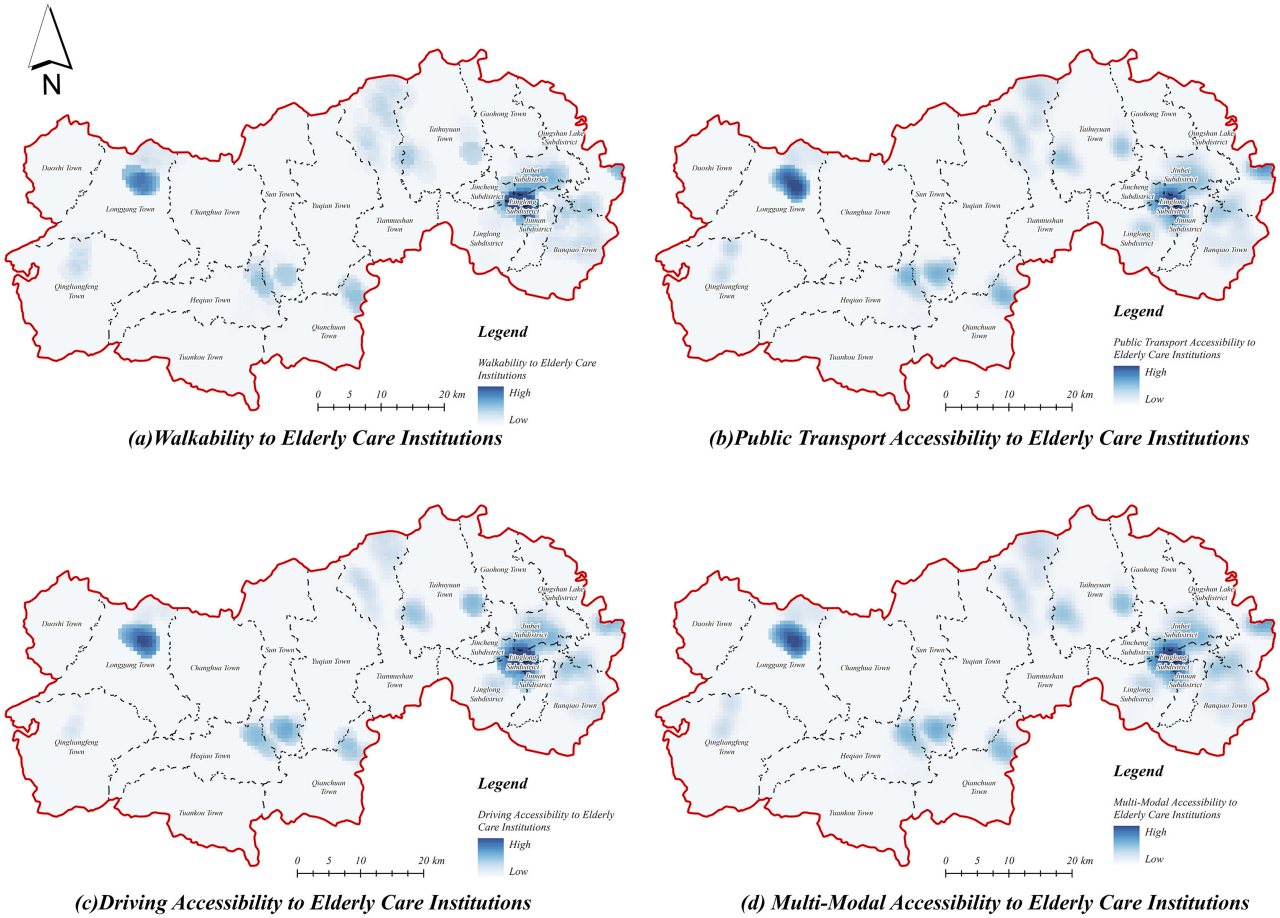
Accessibility of institutions for the older adult. **(a)** Walkability to older adult care institutions. **(b)** Public transport accessibility to older adult care institutions. **(c)** Driving accessibility to older adult care institutions. **(d)** Multi-modal accessibility to older adult care institutions.

The accessibility of older adult care institutions in Lin'an District was assessed using three primary travel modes: walking, public transportation, and driving (Figure 6). The results indicate distinct variations in spatial accessibility, revealing substantial disparities between urban centers and rural areas. Specifically, regions characterized by urban development and mature transportation infrastructure, predominantly located in the eastern and central urban areas, exhibit higher accessibility. Conversely, the peripheral western and central rural areas, which have limited transportation infrastructure and relatively dispersed facility distribution, demonstrate significantly lower accessibility. Among different travel modes, driving accessibility (Figure 6c) clearly exhibits a wider distribution of high-accessibility zones compared to walking and public transportation, thereby significantly improving the overall accessibility of older adult care facilities (Figure 6d).

The accessibility results under the walking mode (Figure 6a) reveal pronounced spatial inequality, with high-accessibility zones significantly constrained by distance, primarily concentrated in urban centers, and sparse or absent in rural regions. Similarly, accessibility through public transportation (Figure 6b) demonstrates notable spatial disparities: although public transit extends accessibility ranges beyond walking, high-accessibility areas remain largely confined to urban cores, highlighting the persistent inadequacy of public transit connectivity in rural areas. In contrast, the driving mode (Figure 6c) substantially expands accessibility coverage, particularly within urban cores, indicating a diminished sensitivity to facility locations and effectively bridging gaps caused by limited transportation infrastructure. Additionally, Longgang Town and other rural townships in the west, which previously displayed low accessibility, benefit considerably from driving mode, reflecting enhanced access under private transport conditions. Moreover, accessibility via driving significantly reduces disparities, underscoring the critical role of multi-modal transportation analyses in accurately capturing the realistic distribution of older adult service accessibility.

#### 3.1.2 Accessibility of home care service centers

**Figure 7:**
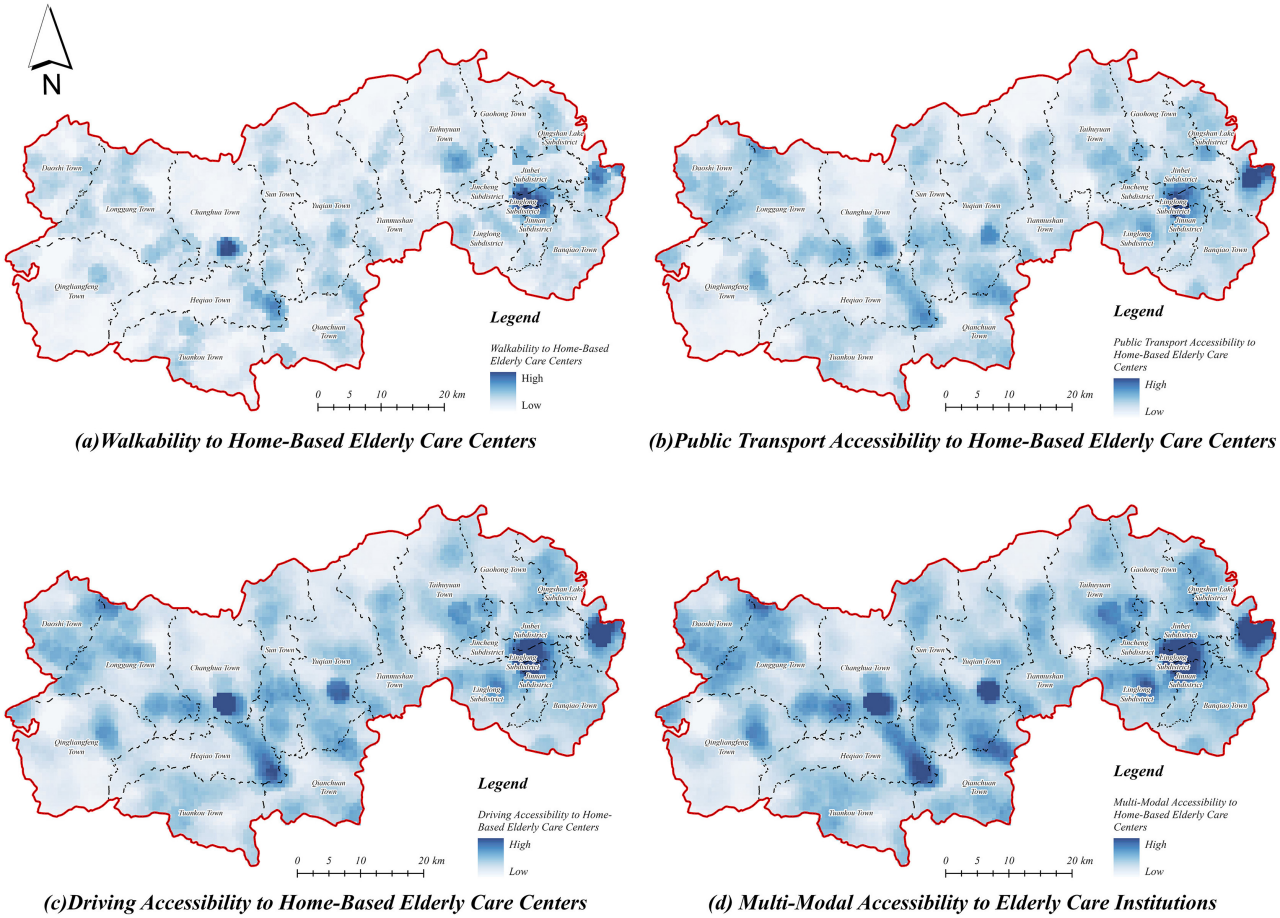
Accessibility of home-based older adult care service centers. **(a)** Walkability to home-based older adult care centers. **(b)** Public transport accessibility to home-based older adult care centers. **(c)** Driving accessibility to home-based older adult care centers. **(d)** Multi-modal accessibility to home-based older adult care centers.

The accessibility of home care service centers in Lin'an District was evaluated through calculations of service coverage areas under different transportation modes (Figure 7). The results indicate relatively balanced overall accessibility across urban and rural areas, characterized by a progressive decrease from the central urban region to peripheral rural townships (Figure 7d). High accessibility values are predominantly observed in the central urban areas, benefiting from higher community density and well-developed transportation infrastructure, whereas rural areas typically present lower accessibility, attributable to dispersed village distributions and inadequate transport infrastructure. Nonetheless, some township centers still exhibit medium to high accessibility values, suggesting localized resource concentration.

Furthermore, the spatial accessibility of home care service centers under walking mode (Figure 7a) exhibits notable spatial heterogeneity, with areas of high accessibility limited by spatial distance, primarily concentrated in the central urban core and selected town centers. Accessibility values sharply decrease outward from these centers, highlighting significant spatial limitations and low coverage in rural regions, particularly in the remote western and northern areas. Under public transportation mode (Figure 7b), accessibility improves modestly compared to walking; however, rural regions, especially remote western and northern areas, continue to experience substantially lower accessibility, revealing deficiencies in public transportation connectivity and infrastructure. In contrast, driving mode (Figure 7c) significantly enhances accessibility, expanding service coverage areas substantially, thereby mitigating spatial disparities. This mode effectively reduces transportation barriers, providing broader and more balanced accessibility for older adult populations across urban and rural contexts.

#### 3.1.3 Accessibility of canteens for the older adult

**Figure 8:**
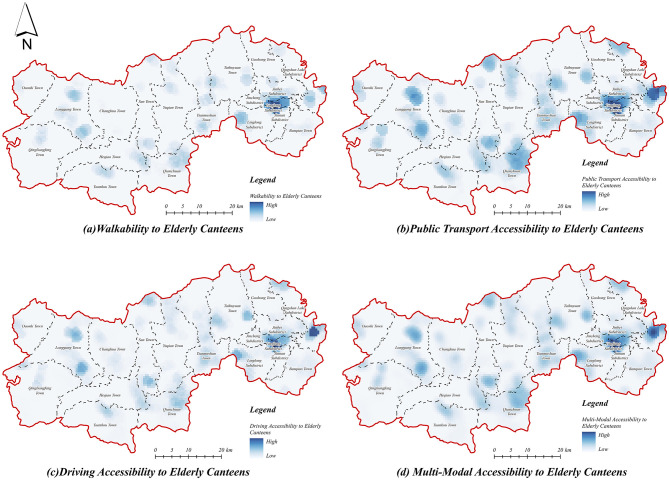
Accessibility of older adult canteen. **(a)** Walkability to older adult canteen. **(b)** Public transport accessibility to older adult canteen. **(c)** Driving accessibility to older adult canteen. **(d)** Multi-modal accessibility to older adult canteen.

The accessibility of senior canteens in Lin'an District was evaluated using multiple transportation modes (Figure 8d). Results indicate generally limited accessibility, characterized by distinct point-like spatial patterns, which highlight notable resource allocation imbalances between urban and rural areas. Areas with high accessibility are primarily concentrated in central urban districts, benefiting from denser facility distributions and developed transportation networks, while rural areas predominantly exhibit lower accessibility values due to dispersed village configurations and limited transportation infrastructure.

In terms of walking accessibility (Figure 8a), senior canteens display a markedly localized spatial clustering, with high-accessibility areas concentrated in the central urban area and township centers, where service demand can be effectively met within a walking threshold. In contrast, rural regions, particularly peripheral zones, exhibit notably lower accessibility, reflecting substantial gaps in service provision and highlighting constraints imposed by spatial distance. Accessibility via public transportation (Figure 8b) demonstrates modest improvements compared to walking, expanding service coverage primarily within central urban and township areas. Nevertheless, rural regions remain underserved, indicating persistent inadequacies in public transportation infrastructure for older adult care service access. Accessibility under the driving mode (Figure 8c) significantly enhances overall coverage, extending medium- and high-accessibility zones into township centers. Despite this improvement, rural areas continue to experience relatively poor accessibility, underscoring persistent limitations due to facility spatial layouts and insufficient distribution density in remote areas.

#### 3.1.4 Comprehensive accessibility of older adult service facilities

**Figure 9:**
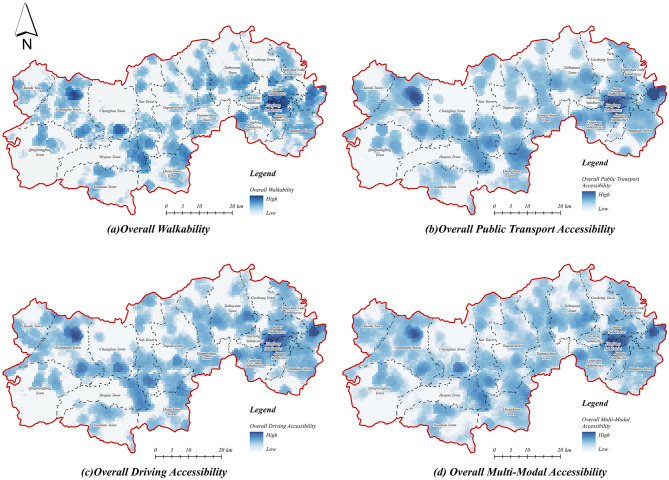
Comprehensive accessibility of older adult service facilities. **(a)** Overall walkability. **(b)** Overall public transport accessibility. **(c)** Overall driving accessibility. **(d)** Overall multi-modal accessibility.

The comprehensive accessibility of older adult service facilities under multi-modal travel (Figure 9d) aggregates accessibility across the three primary travel modes—walking, public transit, and driving—using normalized weights assigned to the three categories of older adult service facilities. The results indicate that accessibility significantly improves under multi-modal travel, revealing a distinct spatial pattern characterized by higher accessibility in central urban areas in contrast to lower accessibility in peripheral rural areas. Specifically, areas with medium and high accessibility are predominantly concentrated in the central city and township centers, whereas remote rural fringe areas continue to exhibit low accessibility. These findings highlight persistent disparities attributable to uneven spatial distributions of older adult care service facilities and variable transportation infrastructure.

Among the individual travel modes, the comprehensive accessibility of older adult service facilities under the walking mode (Figure 9a) exhibits substantial spatial heterogeneity, with high accessibility zones strictly confined to central urban areas and selected township centers, resulting in significant urban-rural discrepancies. The accessibility values rapidly decline from urban centers outward, indicating severe spatial constraints associated with walking travel thresholds. Accessibility via public transportation (Figure 9b) demonstrates improved spatial coverage compared to walking, extending medium- and high-accessibility zones into suburban areas. However, rural accessibility remains limited, underscoring the inadequate capacity and connectivity of public transport infrastructure in remote rural villages. In contrast, driving accessibility (Figure 9c) markedly expands medium and high-accessibility areas, effectively bridging gaps in central urban areas and extending to township centers. Nevertheless, rural regions persistently exhibit low accessibility levels, reflecting enduring limitations due to sparse distribution and uneven allocation of older adult service facilities and limited rural transportation connectivity.

### 3.2 Fairness analysis

#### 3.2.1 Coverage and service supply index analysis

**Figure 10:**
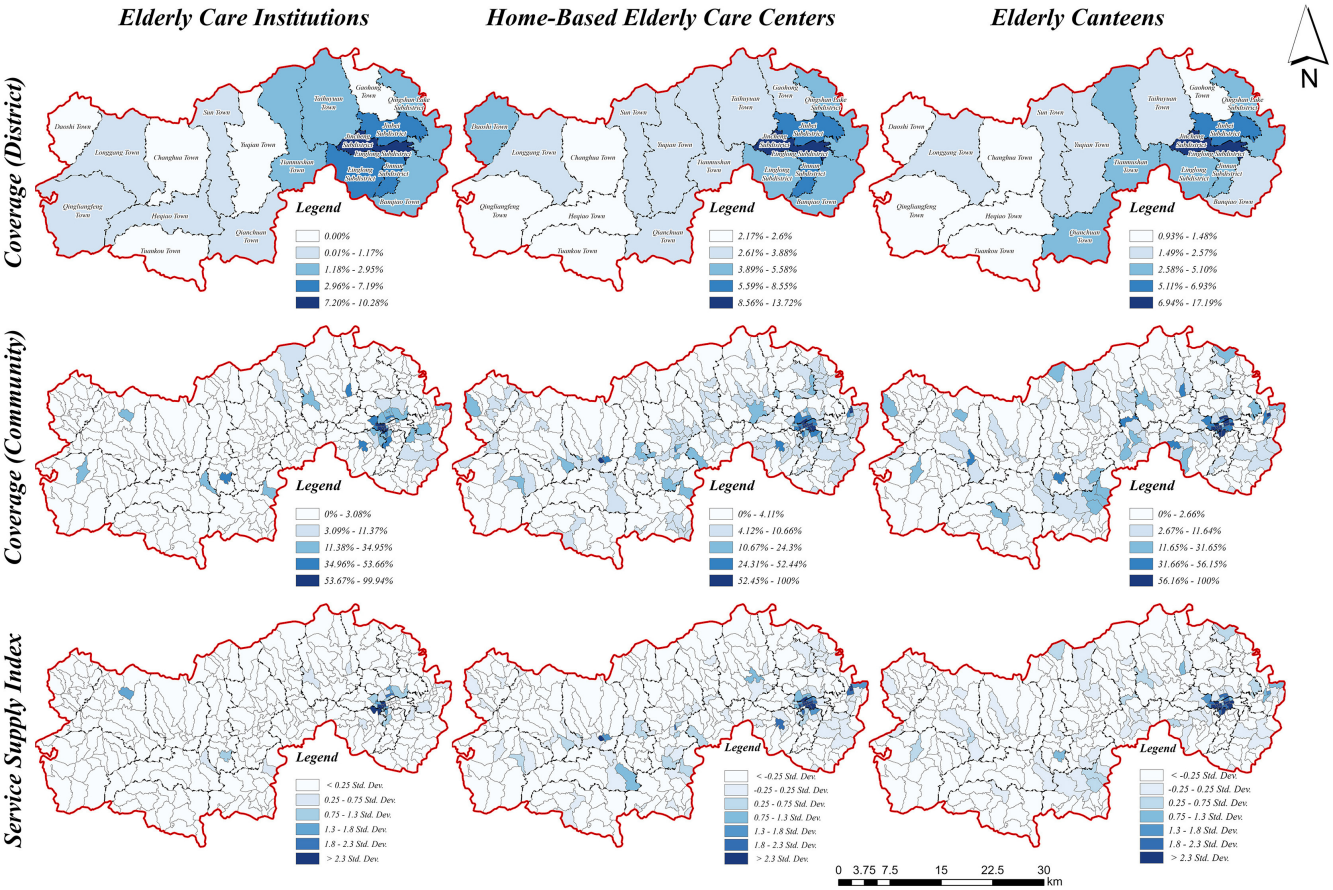
Analysis of coverage and supply index.

Based on the established travel thresholds defined by preliminary investigations, field surveys, and literature reviews, this study determined the walking accessibility coverage of older adult service facilities by generating corresponding buffer zones and evaluated the older adult service supply index through integrating facility coverage with service provision capacities (Figure 10). The results reveal that the walking accessibility coverage and service supply capacities of older adult service facilities exhibit a distinct “core-periphery” spatial structure, characterized by an overall pattern of “high in the east and low in the west.” High coverage rates and robust service supply capacities are primarily concentrated within urban cores and major township centers, benefiting from concentrated population distribution and developed transportation infrastructure. Conversely, rural peripheral areas exhibit significantly lower coverage and weaker service supply, reflecting disparities attributable to facility distribution and limited connectivity.

Significant intra-regional variability exists regarding coverage rates and service supply capacities among different types of older adult service facilities. Specifically, home-based senior care service centers generally demonstrate higher coverage rates and more uniform service supply capacities compared to senior care institutions and senior canteens. Notably, senior canteens exhibit the greatest variability in service supply indexes, indicating substantial resource allocation imbalances and pronounced spatial disparities.

Furthermore, spatial analysis of older adult care institutions (Figure 10) reveals marked disparities: certain rural townships lack any institutional coverage, and extensive rural areas exhibit service supply indexes below 0.25 standard deviations from the mean, indicating severe resource shortages. Conversely, home-based older adult service centers (Figure 10) display comparatively higher overall coverage, reflecting relatively balanced spatial distributions. However, despite better performance than institutional care, disparities persist, especially in remote rural areas, which remain underserved due to inadequate distribution density and limited transportation options.

The coverage rates and service supply capacities of senior citizen canteens (Figure 10) exhibit the most pronounced variability among facility types, closely mirroring the spatial patterns of home-based older adult service centers. In some Urban centers typically reach coverage rates up to 100% and service supply indexes exceeding 2.3 standard deviations above average, whereas rural villages predominantly fall below acceptable coverage thresholds. This distinct urban-rural dichotomy underscores significant inequities, indicating urgent need for policy interventions and optimized resource distribution strategies tailored to the nuanced demands and transportation characteristics of older adult populations in urban-rural integration areas.

#### 3.2.2 Analysis of the Lorenz curve and the Gini coefficient

**Figure 11:**
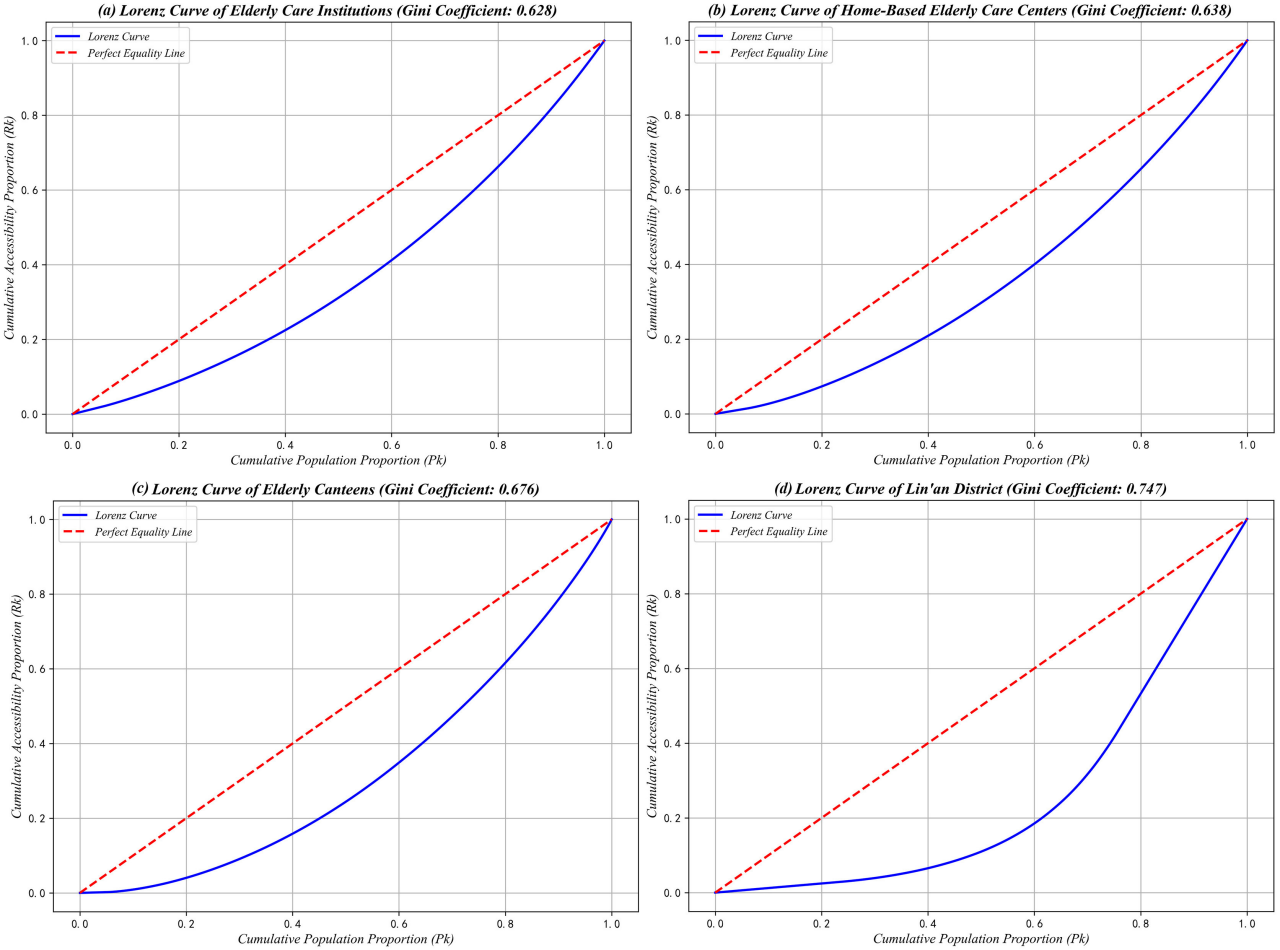
Older adult services facility and Lorenz curve for Linn District. **(a)** Lorenz curve of older adult care institutions (Gini coefficient: 0.628). **(b)** Lorenz curve of home-based older adult care centers (Gini coefficient: 0.638). **(c)** Lorenz curve of older adult canteens (Gini coefficient: 0.676) **(d)** Lorenz curve of Lin'an district (Gini coefficient: 0.747).

Using the Lorenz curve to analyze the accessibility values of different types of older adult service facilities, this study reveals significant disparities and severe mismatches between resource allocation and older adult population distribution in Lin'an District. Specifically, the aggregated results (Figure 11d) indicate that among the 60% of older adult individuals with the lowest per capita accessibility, only 20% of total older adult service resources are accessible, whereas the top 10% with the highest accessibility enjoy 80% of resources, highlighting substantial inequalities in facility allocation.

Further examination of the Lorenz curves for the three types of older adult service facilities demonstrates varying degrees of deviation from the ideal equality line. The distribution patterns for older adult care institutions (Figure 11a) and home-based older adult care service centers (Figure 11b) are relatively similar, both indicating moderate inequities in resource distribution. Specifically, the lowest 60% of the older adult population collectively accesses 40% of resources, whereas the highest 10% accounts for nearly 80%, reflecting a similar degree of spatial concentration for these two facility types.

In contrast, the Lorenz curve for senior canteens (Figure 11c) exhibits a more pronounced deviation from perfect equality, revealing severe inequities in resource distribution. Notably, the lowest 20% of the older adult population has virtually no accessibility to senior canteen services, while the top 10% of the population enjoys around 80% of total resource accessibility, clearly underscoring stark disparities in service provision.

By integrating the Gini coefficient calculations for different types of older adult care facilities, the comprehensive Gini coefficient for older adult service facilities in Lin'an District is determined to be 0.747, indicating substantial imbalances in the overall spatial allocation (Table 6). Furthermore, the Gini coefficients for all individual facility types exceed 0.6, thus classifying their distributions as “extremely inequitable.” Specifically, older adult care institutions, home-based older adult care service centers, and senior canteens all exhibit Gini coefficients above 0.6, signifying pronounced disparities. Among these, older adult care institutions present the lowest coefficient (0.628), suggesting a comparatively more balanced distribution. In contrast, senior canteens display the highest coefficient (0.823), highlighting the most severe spatial inequalities in resource allocation.

**Table 6:** Gini coefficient of older adult care service facilities and Lin'an District.

**Type/ region**	**Older adult care institution**	**Home care service center**	**Older adult canteen**	**Lin'an District**
***Gini index***	*0.628*	*0.638*	*0.676*	*0.747*

These findings collectively demonstrate that the distribution of older adult care resources in Lin'an District is notably uneven across facility types, with senior canteens exhibiting particularly pronounced spatial imbalances. Consequently, this pattern underscores the urgent necessity for differentiated optimization strategies tailored to specific facility types, aimed at improving the equity and effectiveness of older adult service resource allocations between urban and rural areas.

### 3.3 Analysis of urban-rural disparities

**Figure 12:**
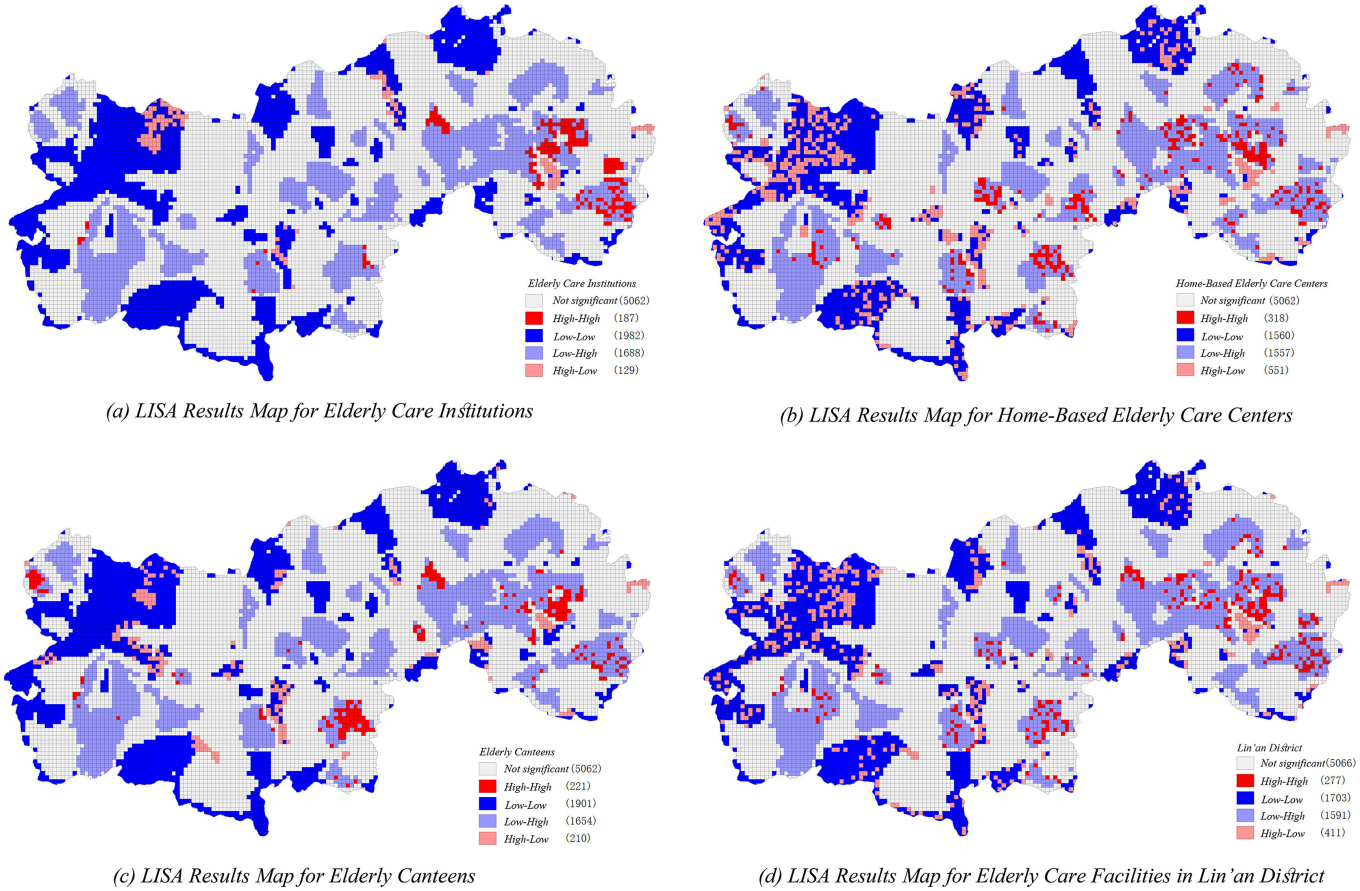
Bivariate LISA analysis of older adult service facilities and Linn district figure. **(a)** LISA results map for older adult care institutions. **(b)** LISA results map for home-based older adult care centers. **(c)** LISA results map for older adult canteens. **(d)** LISA results map for older adult care facilities in Lin'an distrcit.

This study utilized GeoDa 1.22 software to calculate the Moran's Index, quantitatively examining spatial autocorrelation and assessing urban-rural disparities in accessibility across various types of older adult service facilities within Lin'an District (Figure 12). Results from the bivariate local spatial autocorrelation analysis (Moran's I) (Figure 12d) reveal significant spatial heterogeneity in facility accessibility between urban and rural areas. The identified “high-need-high-allocation” clusters are mainly concentrated in the core of the central urban area and select township centers, indicating a strong alignment between older adult service facilities and the older adult population distribution. Conversely, rural areas generally display lower accessibility values, though some township centers, such as Longgang, exhibit relatively adequate alignment between resource allocation and local demand. Nevertheless, spatial mismatches remain pronounced in certain peripheral townships.

Spatial clusters categorized as “high-demand-low-allocation” and “low-demand-high-allocation” primarily emerge at the peripheries of the central urban area and in several township boundaries. These spatial patterns highlight pronounced discrepancies and inefficient resource allocations, emphasizing the complexity of matching older adult service provision with localized population demands.

Among different older adult service facility types, senior citizen canteens (Figure 12c) display the most spatially concentrated hotspot distributions and relatively limited cold-spot coverage, suggesting a more balanced spatial layout in rural areas compared to other facility types. Conversely, home-based older adult service centers (Figure 12b) exhibit greater spatial heterogeneity, with a notable concentration of mismatched allocation, indicating insufficient service supply in rural regions. Older adult care institutions (Figure 12a) display spatial hotspots primarily within urban cores, reflecting higher resource availability and service supply concentration within urban contexts. Additionally, a considerable number of non-significant areas are identified across all facility types, suggesting that significant proportions of older adult populations remain underserved due to suboptimal facility distribution and uneven spatial resource allocations.

## 4 Discussion

### 4.1 Potential application of an improved two-step mobile search method incorporating real-time travel data

Potential application of an improved two-step mobile search method incorporating real-time travel data

This study refined and analyzed the accessibility and spatial distribution patterns of older adult service facilities between urban and rural areas in Lin'an District by introducing an enhanced 2SFCA method and integrating real-time travel data from AMap's open API. The results provide a detailed characterization of accessibility disparities, revealing distinct spatial differences in older adult service resource allocation.

In particular, the eastern central urban areas exhibit higher accessibility, benefiting from advanced economic development, high population density, and well-established transportation infrastructure, thus achieving relatively equitable service resource allocation. This finding is consistent with results reported by Li et al. (11), who similarly emphasized the advantageous impact of urban centralization on institutional accessibility. However, previous studies employing the potential model (11) or traditional 2SFCA (30) have largely overlooked dynamic variations resulting from multi-modal travel conditions and real-time traffic fluctuations. Addressing these methodological limitations, the present study employs an enhanced 2SFCA by integrating real-time travel data from the AMap API. Consequently, this method offers a more dynamic, detailed, and precise spatial accessibility evaluation.

The incorporation of real-time travel data into accessibility analysis has enabled a more accurate representation of older adult service resource distribution and access disparities, effectively capturing the impacts of transportation dynamics on accessibility. This methodological advancement not only provides more realistic assessments, but also facilitates detailed spatial differentiation within accessibility analyses. For instance, the improved method identifies accessibility variations previously obscured by traditional approaches, such as significant accessibility enhancements in urban-rural transitional areas under driving mode, which traditional models failed to reveal. Furthermore, this study's findings corroborate the conclusions of Li et al. (11), confirming that economically developed regions with better transport infrastructure tend to exhibit higher resource equity, yet offering a more comprehensive and dynamic perspective through real-time data integration.

### 4.2 Analysis of the mechanisms affecting the mismatch between supply and demand

The findings of this study indicate that the mismatch between the supply and demand for older adult care facilities in Lin'an District results from complex interactions among multiple factors, including the spatio-temporal dynamics of older adult populations, transportation accessibility, geographical constraints, and governmental policies, collectively influencing the efficiency and equity of resource allocation for older adult care services.

From the demand perspective, the spatio-temporal distribution of older adult populations directly determines the intensity and spatial configuration of older adult care services. The older adult population in Lin'an District exhibits a clear concentric distribution pattern, with significant concentrations in economically developed urban cores and selected remote villages, which aligns with previous research findings (31, 32). Additionally, the migration of younger populations toward central urban areas has accelerated aging in rural regions. Although these rural areas typically have lower overall population densities, their disproportionately higher aging rates, coupled with inadequate older adult care facility scale and capacity, exacerbate supply-demand imbalances, resulting in substantial resource deficiencies.

The demographic disparities identified are further intensified by transportation accessibility and geographical conditions. In urban areas, advanced transportation infrastructure and extensive public transit networks significantly enhance older adult populations' access to care facilities. In contrast, suburban and rural regions experience reduced accessibility and restricted older adult mobility due to limited transportation infrastructure, corroborating previous studies emphasizing the pivotal role of transportation in shaping public service accessibility (33, 34). Additionally, the complex geographical conditions of Lin'an District, characterized by mountainous terrain and intricate river networks, further constrain effective service coverage and complicate facility allocation strategies.

Beyond demographic and geographical constraints, governmental policies and planning standards significantly impact the spatial layout of older adult care facilities. The Zhejiang provincial government actively promotes an integrated “medical-nursing combination and intergenerational embedded care” model aimed at enhancing service comprehensiveness and diversity. However, current construction standards lack dynamic adjustment mechanisms, limiting the flexibility of facilities in adapting to evolving older adult care needs. Although a certain number of older adult care facilities have been established in rural regions, their practical effectiveness remains constrained by inadequate integration with medical and childcare resources, leading to lower utilization rates. This observation aligns with previous research (35), while the present study further highlights specific implementation shortcomings, such as insufficient incorporation of healthcare and nursing functions, ultimately hindering the effective realization of integrated older adult care models.

### 4.3 Strategies for optimizing the layout of older adult facilities based on equity

Addressing the uneven distribution of older adult care facilities necessitates differentiated zoning strategies tailored to the spatial heterogeneity of older adult population densities, thereby optimizing resource allocation. Practical experiences from various cities demonstrate that adopting population-density-based differentiated strategies significantly enhances the equity and accessibility of older adult care services. For instance, Beijing successfully improved resource equity through its “Special Plan for Older adult Facilities,” which employed targeted allocation strategies aligned with older adult population densities. Similarly, New York City utilized spatial-temporal analyses of older adult population dynamics to rationally allocate services, effectively mitigating resource concentration and wastage. These examples highlight the critical role of localized differentiation in older adult service planning (36). Drawing upon such experiences, our study suggests prioritizing facility construction or upgrading in areas characterized by insufficient resources and poor accessibility, particularly in communities exhibiting high older adult concentrations but inadequate service provisions. To facilitate practical implementation, local governments should establish dedicated funding mechanisms supporting facility development in underserved areas and actively encourage public-private partnerships (PPPs) to expand service delivery capacities. Additionally, promoting decentralized distributions of higher-level older adult care facilities will improve overall coverage and equity, preventing excessive resource centralization and achieving a more balanced alignment between supply and demand.

The effectiveness of optimized facility allocation also critically depends on complementary improvements in transportation infrastructure, especially through the enhancement of public transit systems at township and community levels. Existing research underscores the strong dependence of older adult populations on public transportation, highlighting that improved connectivity between service facilities and older adult users substantially increases accessibility and travel efficiency (37). For example, Vienna effectively improved older adult residents' accessibility and travel satisfaction by optimizing bus routes that connect residential areas with public amenities such as green spaces. Building on these international experiences (30, 38), this study employs real-time travel data sourced from open mapping platforms to propose targeted transportation optimization strategies for older adult-concentrated regions, aiming to enhance transit connectivity through scientifically designed public transit routes. To precisely align transportation improvements with older adult service demands, local governments should establish effective coordination mechanisms between transportation and social service planning departments and provide financial incentives for transport providers to establish older adult-friendly transit routes and shuttle services. This approach aims to minimize travel barriers, enhance service accessibility, and foster greater spatial equity in older adult care provision.

Alongside spatial optimization of facilities and transportation enhancements, digital technologies should be leveraged to establish a dynamic “monitoring-assessment-adjustment” mechanism, facilitating flexible adaptation to older adult populations' diverse and evolving needs. For instance, Zhejiang Province innovatively implemented the “Happy Older adult at Home” model, integrating home-based older adult care, community institutions, and intelligent digital older adult-care platforms. By continuously monitoring demographic shifts and service demands, this model dynamically adjusts resource allocations and service provisions, significantly enhancing responsiveness and efficiency. This underscores digital technology's transformative role in achieving adaptive and responsive older adult care services. In contrast to traditional static planning methodologies, this research emphasizes the establishment of real-time, dynamic management mechanisms to continuously monitor population changes and evolving demands, enabling timely adjustments in facility locations, service capacities, and resource distributions. To further enhance effectiveness, multi-stakeholder governance frameworks involving governments, technology companies, and community-based organizations should be established, fostering collaborative data-sharing and decision-making. Moreover, promoting multi-functional facility designs and diversified service offerings will proactively accommodate the shifting and varied needs of older adult populations, ultimately creating a more inclusive, equitable, and resilient older adult care ecosystem.

### 4.4 Research limitations and future perspectives

This study evaluated older adult care facility accessibility by employing buffer zone analysis, yet several methodological limitations emerged from this approach. Although buffer zones offer a straightforward means of measuring service coverage, they cannot adequately represent older adult travel behaviors and their variations across different transportation modes, complicating efforts to establish universally applicable accessibility thresholds. Such limitations may introduce biases and reduce the accuracy of the evaluation. The current analysis was restricted to three conventional travel modes—walking, public transportation, and driving—thus excluding increasingly prevalent alternatives, such as electric bicycles. Omitting these emerging transportation modes could potentially weaken the comprehensiveness and generalizability of the accessibility assessment. Meanwhile, reliance on open-source real-time traffic data from platforms like AMap introduced potential biases related to data quality, accuracy variations, and calibration uncertainties, all of which could undermine the robustness of the analysis.

Another significant constraint involves the temporal dimension of the accessibility evaluation, which focused exclusively on a single time point. Given that older adult facility accessibility dynamically evolves over time in response to changing demographics, policy interventions, and infrastructure development, analyzing accessibility at only one temporal snapshot cannot fully capture these ongoing trends or reveal longitudinal variations. The dependence on publicly accessible data (such as AMap, POI, and AOI datasets), characterized by potential inaccuracies or inconsistencies, further challenges the precision and reliability of the findings, potentially limiting their wider applicability.

To effectively overcome these methodological shortcomings, future research may incorporate advanced behavioral-spatial simulation models capable of accurately depicting older adult travel patterns and mode-specific accessibility scenarios, including emerging modes like electric bicycles. Employing sophisticated platforms, such as AnyLogic software, would allow researchers to simulate realistic older adult mobility patterns and thereby achieve more precise alignment between service facilities and diverse older adult mobility demands (39). Additionally, the integration of continuous multi-temporal real-time traffic flow analytics and big data techniques could greatly enrich the granularity and comprehensiveness of accessibility analyses, allowing more nuanced evaluations of accessibility dynamics across different spatial and temporal scales (21). Recognizing the increasing complexity and diversification of older adult populations, future studies should also explore differentiated demand models tailored to age, gender, geographical, and cultural contexts (40). By systematically incorporating digital technology, real-time monitoring, and multi-stakeholder governance frameworks involving local governments, private enterprises, and community organizations, future research can significantly improve the responsiveness and adaptability of older adult service provision, ultimately contributing to the development of a more inclusive and resilient older adult care ecosystem.

## 5 Conclusion

This study systematically evaluated the spatial accessibility and equity of older adult service facilities in Lin'an District by employing an improved multi-modal 2SFCA method incorporating Gaussian decay functions. The results revealed significant spatial disparities and prominent mismatches between supply and demand at the community level. Accessibility exhibited notable spatial heterogeneity, characterized by a clear “high in the east and low in the west” distribution, reflecting a distinctive core-periphery spatial structure. Central urban areas benefited from a concentration of facility resources, whereas rural and peripheral regions experienced insufficient service coverage and substantial equity deficiencies. These findings emphasize the necessity of formulating more precise and equitable planning strategies, particularly targeting underserved rural communities.

To address these issues and advance methodological rigor, future research should prioritize the following directions: (1) integrating diverse, multi-source datasets—including socio-economic characteristics, real-time mobility patterns, and older adult activity behaviors—to provide a deeper understanding of dynamic service demands and their spatiotemporal dynamics; (2) refining analytical methods by incorporating dynamic transportation networks, multi-modal travel analyses, and advanced personalized demand-service matching techniques, thereby enhancing the accuracy and adaptability of accessibility models; and (3) expanding research perspectives beyond urban-rural integrated contexts, conducting cross-regional comparative studies to formulate flexible facility allocation strategies that account for varying geographical, demographic, and socio-economic conditions.

## Data Availability

The datasets presented in this study can be found in online repositories. The names of the repository/repositories and accession number(s) can be found in the article/supplementary material.
